# Altered awareness of action in Parkinson’s disease: evaluations by explicit and implicit measures

**DOI:** 10.1038/s41598-017-08482-0

**Published:** 2017-08-14

**Authors:** Naho Saito, Keisuke Takahata, Hodaka Yamakado, Nobukatsu Sawamoto, Satoshi Saito, Ryosuke Takahashi, Toshiya Murai, Hidehiko Takahashi

**Affiliations:** 1 Department of Psychiatry, Kyoto University Graduate School of Medicine, Kyoto, Japan; 2 Department of Functional Neuroimaging Research, National Institute of Radiological Sciences, Chiba, Japan; 3 Department of Neurology, Kyoto University Graduate School of Medicine, Kyoto, Japan; 4 Department of Human Health Sciences, Kyoto University Graduate School of Medicine, Kyoto, Japan

## Abstract

Deficits in the integration of motor prediction and its feedback have been reported in Parkinson’s disease. Conscious awareness of action is proposed to emerge under the integration of motor prediction and its feedback. Thus, it may lead to changes in the awareness of the authorship of action (in other words, the sense of agency) in Parkinson’s disease. We have employed both explicit and implicit measures to assess the awareness of action in Parkinson’s disease and matched controls. As an explicit measure, an action recognition task requiring explicit judgments was used. Patients showed less attribution of their movements to non-biased and angular-biased visual feedbacks. As an implicit measure, the temporal attraction between the perceived time of actions and their effects, which is known as intentional binding task, was used. While action-effect association was observed in the control group, actions were not experienced as having shifted towards their subsequent effects in the patient group. These tendencies were consistent regardless of the side of the asymmetrical motor symptoms. These results may reflect an underlying abnormality in the awareness of voluntary action in Parkinson’s disease.

## Introduction

Parkinson’s disease (PD) is a progressive neurological disorder characterized by the degeneration of dopamine-producing neurons. It is the second most common neurodegenerative disorder after Alzheimer’s disease, and its incidence range from 1.5% to 2% after age 65^1^. Motor symptoms develop in an asymmetrical order, with symptom dominance either on the right or left side^2^. Besides motor symptoms, patients experience various deficits in the emotional and cognitive domain, as well as in sensorimotor integration^3–5^.

The belief that we ourselves are responsible for our own action outcome has been referred to as the “awareness of action” or the “sense of agency”. It is one of the minimal components of self-awareness known as “minimal self”, that is “a consciousness of oneself as an immediate subject of experience”^6^. The concept is related to some common neurocognitive models and neural processes^6^. Disruptions in the awareness of action present widely among psychiatric and neurological diseases^7–9^. Growing evidence points to the linkage between disruptions in the awareness of action and movement disorders, where different aspects of volitional control have been focused^10–14^. It has been suggested that the conscious awareness of action emerges under the integration of motor prediction and its feedback in the framework of the central monitoring theory^15, 16^. Comparison of prediction with sensory afference will enable us to distinguish self-produced sensory information from externally caused events. In addition to this predictive process, the awareness of action is also known to be affected postdictively^17, 18^. That is, experiences of causation for voluntary actions are determined in part by post-action information^19^. Various cues in the level of perception, cognition and emotion are known to contribute to the awareness of action^20^.

Previous studies have approached the awareness of action by two distinct methods. One classical approach is an action recognition task requiring explicit judgments. Participants are given visual feedback of a voluntary action, which is either neutral or distorted. They are asked if their action is responsible for the given feedback, in a way that the feedback is either in concordance with their action or not^21^. The other approach is to measure the awareness of action implicitly as an attraction between the perceived time of actions and their effects^22^. This is called the intentional binding effect. Participants view a clock hand rotating over a certain period of time, and make a volitional button press at the timing of their own choosing, as in Libet’s paradigm^23^. The button press will be followed by a tone as its consequence. Perception of actions will be shifted towards the subsequent effects (action binding: AB), and that perception of effects will be shifted towards the preceding actions (tone binding: TB). Because such action-effect association occurs only when the actions are made voluntarily but not when they are made involuntarily, this has been suggested as being a quantitative marker for measuring awareness of action^22^. These two empirical methods have been repeatedly verified through many studies^12, 19, 24–30^, and the two approaches have been linked to the implicit “feeling of agency” and the explicit “judgement of agency”, respectively^31^. The lower level feeling of agency represents a step that is not conceptual upon our actions. The second level judgement of agency represents a conceptual, interpretative step of making judgement for attribution of actions. The dissociation of these two aspects has been reported both theoretically^31^ and experimentally^32^.

Given the disruption of sensorimotor integration in PD^3–5^, it has been questioned whether there are subsequent changes in the awareness of action in PD. Being able to experimentally verify the theoretical framework of central monitoring is one of the advantages for testing the issue in PD patients, because there have been accumulative studies on sensorimotor integration in PD. From a clinical viewpoint, PD patients manifest various psychiatric disturbances including anxiety, apathy and depression^33^. Patients experience constant frustration in unreliable motor execution^34^. Uncertainties in the activities of daily life cast a negative effect on patients’ quality of life^33, 34^. It would be of clinical importance to know if patients are experiencing an uncertainty in the awareness of action even for their intended actions.

A study has measured intentional binding in PD patients, comparing them when they are on and off dopaminergic medication^27^. PD patients on medication showed an increase in the overall amount of action-effect association compared to those off medication. However, it has not been clear whether this result following dopamine intake was driven by improvements in central sensorimotor processing of the disease or by secondary psychological changes following improvements in motor impairment. The authors of the study suggested the possibility of the former, although no direct test was performed. Motor impairment may cause altered visual feedback as a secondary effect. The knowledge of having motor disabilities may as well influence priors and beliefs of action. It is known that priors and beliefs of action influence the experience of personal causation^35, 36^. Additionally, there is a “self-serving” cognitive bias for underestimating the awareness of action for negative outcomes^37^. These may also be factors to affect the awareness of action^20^. It has remained unclear whether the primary components of central sensorimotor processing or secondary psychological effects from motor impairment matter the most.

The aim of our study was to determine whether there is a change in the awareness of action in PD patients, and to detect whether the change is driven primarily by deficits in central sensorimotor processing or secondary by motor impairment. We have tested two different explicit and implicit measures, based on the dissociations between explicit and implicit aspects in awareness of action. More specifically, we focused on the asymmetrical motor symptoms in PD, conducting measures with the hands of both the more affected side and the less affected side of motor symptoms. If the awareness of action changes in PD would be more related to the deficits in primary components of central sensorimotor processing, which have been shown to be evident prior to the appearance of motor signs^38, 39^, the results between the two sides would not differ. If the awareness of action changes in PD would be related to the secondary effects from motor impairment, there could be a difference in the results between the two sides. This asymmetrical comparison will be one of the advantages for investigating the awareness of action in PD, compared to other pathologies, that volitional control of action would be affected in a symmetrical manner.

## Results

### Demographic and clinical characteristics

Demographic and clinical variables are presented in Table 1. The average Unified Parkinson’s Disease Rating Scale (UPDRS) right/left subscale of the more-affected side was 10.2 (SD: 4.6), and of the less-affected side was 6.3 (SD: 3.7). There was a significant difference between the scores of the two sides [*t*(18) = 4.69, *p* < 0.001].

**Table 1:** Demographic and clinical variables.

	Parkinson’s disease patients (n = 19)		Controls (n = 25)		Analysis
	mean	SD	mean	SD	*p*
**Demographic scores**					
Age (years)	66.0	6.2	64.9	2.9	0.49
Years of education	13.7	2.2	13.9	2.2	0.79
Total daily LED (mg/day)	369.5	276.5	—	—	—
**Clinical scores**					
BDI	14.7	8.8	9.3	6.5	0.02
Apathy Scale	13.9	6.5	11.7	5.1	0.20
General Self Efficacy Scale	75.7	16.1	81.6	11.7	0.17
Cognistat	96.5	4.6	98.4	4.2	0.16
FAB	15.1	2.3	15.3	1.7	0.77
UPDRS motor subscale	23.2	9.4	—	—	—

Statistics of demographic and clinical variables in PD and control groups are shown.

### Experiment 1- implicit task

#### Baseline condition

To determine whether there was a difference in time perception between groups, the results of the baseline condition were analyzed. Perception of action alone when the action was made by right and left hand did not differ in controls [*t*(24) = 1.26, *p* = 0.22]. Perception of action alone when the action was made by the more and the less affected hand did not differ in patients [*t*(18) = 0.33, *p* = 0.75]. Therefore, the average value of the perception of action when made by both hands was used in the following analysis. The perception of action alone between patients and controls did not differ [*t*(42) = 0.40, *p* = 0.69]. The perception of tone alone between patients and controls also did not differ [*t*(42) = 0.09, *p* = 0.93]. The average number of unsuccessful trials in baseline condition was 0.05 per block in patients, and 0 per block in controls.

#### Agency condition

In controls, a positive shift in the perceived time of action was observed in the agency condition compared to the baseline condition (AB) [right: 64.2 (SD: 119.4) ms, *t*(24) = 2.69, *p* = 0.013; left: 78.3 (SD: 117.7) ms, *t*(24) = 3.32, *p* = 0.003]. At the same time, a negative shift in the perceived time of tone was observed in the agency condition compared to the baseline condition (TB) [right: −113.1 (SD: 155.5) ms, *t*(24) = 3.64, *p* = 0.001; left: −114.5 (SD: 171.3) ms, *t*(24) = 3.34, *p* = 0.003].

**Figure 1:**
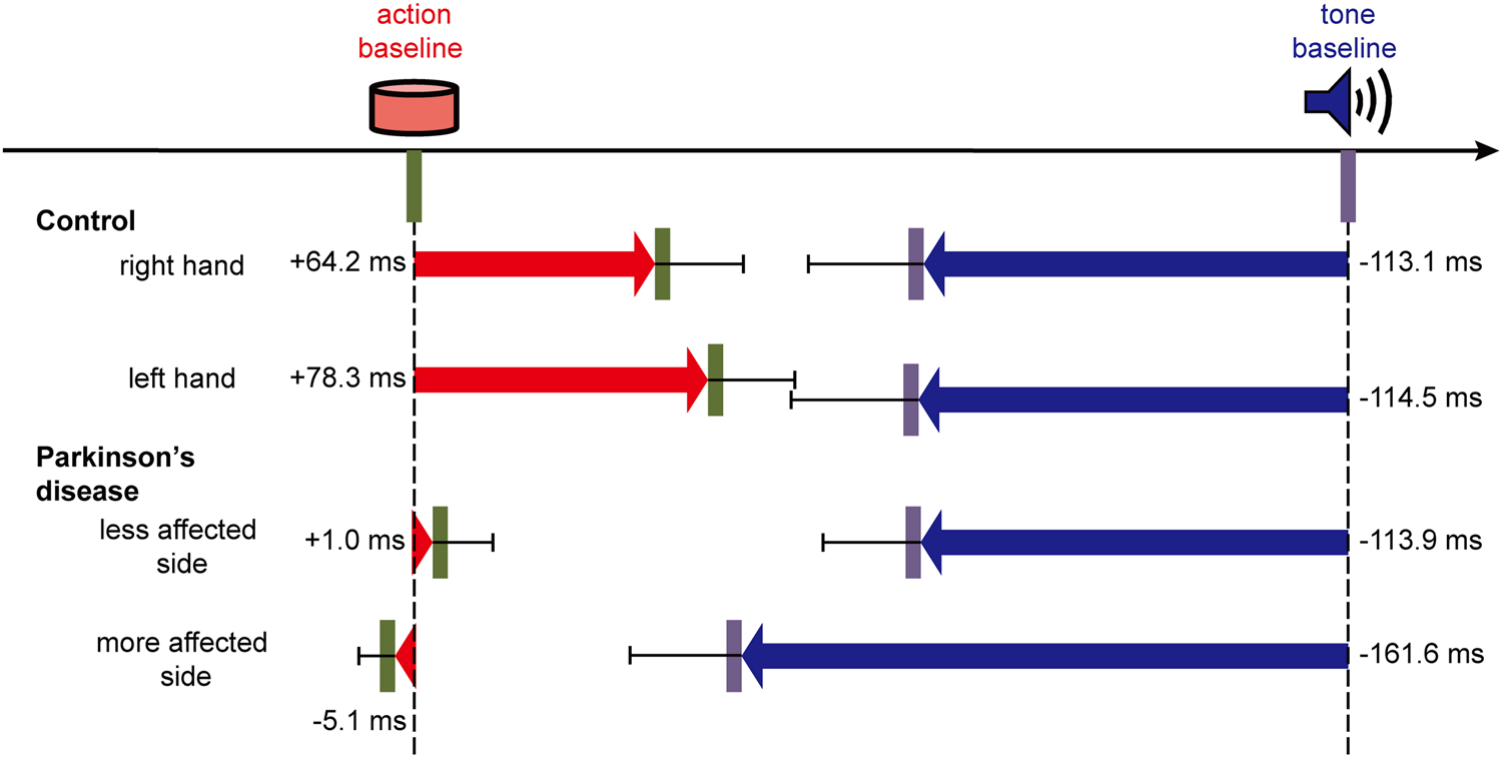
Perceived times of actions and tones in controls and in PD group. Red arrows represent perceived timing of actions, and blue arrows represent perceived timing of tones in agency condition compared to baseline condition. Bars represent standard errors.

In PD patients, on the other hand, this shift of perceived time of action was not observed in the agency condition compared to the baseline condition (AB) [less affected side: 1.0 (SD: 71.6) ms, *t*(18) = 0.06, *p* = 0.95; more affected side: −5.1 (SD: 43.8) ms, *t*(18) = 0.50, *p* = 0.62]. As for tone, a negative shift in the perceived time was observed in the agency condition compared to the baseline condition (TB) [less affected side: −113.9 (SD: 115.0) ms, *t*(18) = 4.32, *p* < 0.001; more affected side: −161.6 (SD: 131.2) ms, *t*(18) = 5.37, *p* < 0.001] (Fig. 1).

AB was analyzed by mixed-effect ANOVA, with group (patients versus controls) as between-subject factor, and hand (more-affected versus less-affected in patients, left versus right in controls) as within-subject factor. This revealed a main effect of group [*F*(1,42) = 6.60, *p* = 0.01]. Neither an interaction of group × hand [*F*(1,42) = 1.44, *p* = 0.24], nor a main effect of hand [*F*(1,42) = 0.23, *p* = 0.64] was observed. TB was also analyzed by mixed-effect ANOVA, with group (patients versus controls) as between-subject factor and hand (more-affected versus less-affected in patients, left versus right in controls) as within-subject factor. This revealed no interaction of group × hand [*F*(1,42) = 3.45, *p* = 0.07], main effect of group [*F*(1,42) = 0.31, *p* = 0.58], or main effect of hand [*F*(1,42) = 3.87, *p* = 0.06]. The average number of unsuccessful trials in agency condition was 0.07 per block in patients, and 0.03 per block in controls.

No significant correlation was found by Spearman’s rank correlation analysis between patients’ scores of the two conditions (AB and TB averaged across hands) and age, Unified Parkinson’s Disease Rating Scale (UPDRS) motor scores, total daily levodopa equivalent dose (LED), Beck Depression Inventory (BDI), Apathy Scale, General Self Efficacy Scale and Neurobehavioral Cognitive Status Examination (Cognistat) total scores. No significant correlation was also found between controls’ scores of the two conditions (AB and TB averaged across hands) and age, BDI, Apathy Scale, General Self Efficacy Scale and Cognistat total scores. The statistical values for correlation are shown in Supplementary Table S1.

### Experiment 2- explicit task

**Figure 2:**
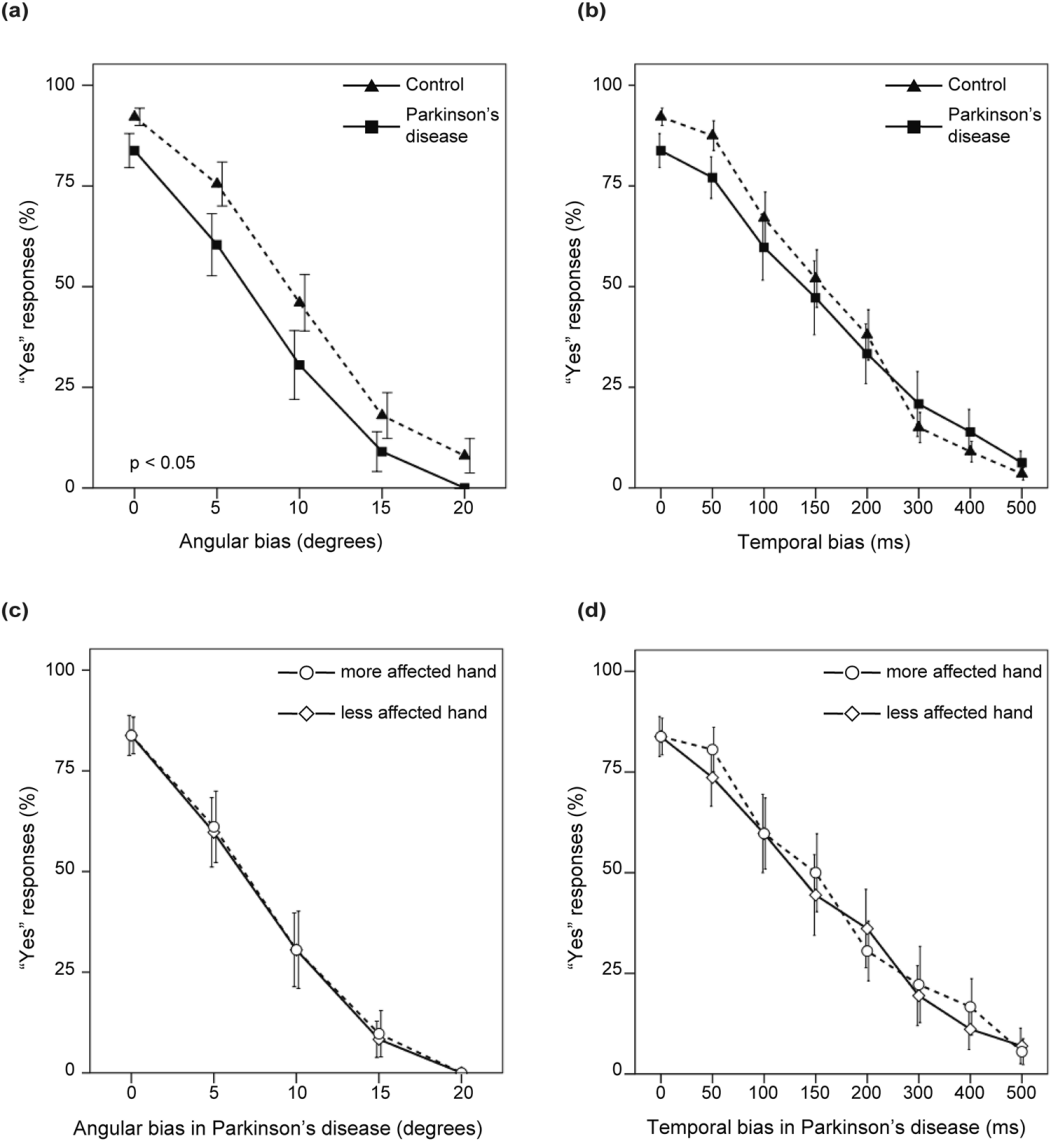
“Yes” responses for judgements in relation to the movements on screen with their actual movements. Bars represent standard errors.

In both patients and controls, the most “yes” responses were given in the neutral condition, with the percentage of “yes” responses decreasing as the angular or temporal biases increased. Angular biases condition was analyzed by mixed-effect ANOVA, with group (patients versus controls) as between-subject factor, and event (gap of 0°, 5°, 10°, 15°, or 20°) and hand (more-affected versus less-affected in patients, left versus right in controls) as within-subject factor (Fig. 2). There was a main effect of group [*F*(1,41) = 4.31, *p* = 0.04] and a main effect of event [*F*(2.9,118.1) = 120.7, *p* < 0.001]. There were no significant interactions of event × group × hand [*F*(2.3,93.5) = 0.82, *p* = 0.46], event × group [*F*(2.9,118.1) = 0.34, *p* = 0.79], group × hand [*F*(1,41) = 0.89, *p* = 0.35], event × hand [*F*(2.3,93.5) = 0.60, *p* = 0.57] or main effect of hand [*F*(1,41) = 0.48, *p* = 0.49]. Temporal biases condition was also analyzed by mixed-effect ANOVA, with group (patients versus controls) as between-subject factor, and event (gap of 0, 50, 100, 150, 200, 300, 400, or 500 ms) and hand (more-affected versus less-affected hand in patients, left versus right hand in controls) as within-subject factor. There was a main effect of event [*F*(3.4,138.9) = 117.9, *p* < 0.001]. There were no significant interactions of event × group × hand [*F*(4.6,187.3) = 0.49, *p* = 0.77], event × group [*F*(3.4,138.9) = 1.16, *p* = 0.33], group × hand [*F*(1,41) = 1.36, *p* = 0.25], event × hand [*F*(4.6,187.3) = 1.01, *p* = 0.41], main effect of group [*F*(1,41) = 0.24, *p* = 0.63], or main effect of hand [*F*(1,41) = 0.08, *p* = 0.78]. There was no unsuccessful trial in the two groups.

No significant correlation was found by Spearman’s rank correlation analysis between patients’ total number of “yes” responses in the two conditions (with angular and temporal biases) and age, UPDRS motor scores, total LED, BDI, Apathy Scale, General Self Efficacy Scale and Cognistat total scores. Significant correlation was also not found between controls’ total number of “yes” responses of the conditions and age, BDI, Apathy Scale, General Self Efficacy Scale and Cognistat total scores. The statistical values for correlation are shown in Supplementary Table S1.

## Discussion

Our study investigated the awareness of volitional action in PD through explicit and implicit measures. Both explicit and implicit measures were assessed, because differences between the two aspects have been pointed out^32, 40^. In the implicit task, intentional binding effects were confirmed in our control group. In the PD group, AB was not observed while TB was observed. One plausible cause for this could be the change of time perception in PD patients. However, the perception of action alone and tone alone in the baseline condition did not differ between patients and controls, suggesting that time perception itself was preserved in our patient group. Another cause could be the problem of attention in patients. Regarding this point, TB did not differ between controls and the PD group, suggesting that it did not simply stem from impaired allocation of attention to action and tone.

In the intentional binding paradigm, different explanations for AB and TB have been proposed^17^. It has been suggested that AB, but not TB, results from cue integration, where the sensorimotor system combines information from various sources to integrate prediction and action consequences^28^. As uncertainty about the action effect increases, action binding will be reduced^28^. Because patients are nervous about their unreliable motor execution^34^, these uncertainties for action outcomes might have led to the diminishment of AB, together with the reported deficits in sensorimotor integration^3–5^. TB is explained as being driven by a different mechanism, in line with perceptual latencies^28, 41^. This may be the reason for the different results for AB and TB in PD patients.

It has also been shown that AB depends on postdictive as well as predictive processes^17^. The contents of the observed outcome influence the agent retrospectively for ownership of the effect^18^. Intentional binding is known to change according to the contents of the effects; action-effect association will be weakened for negative outcome variance^19, 29^. Action is known to be linked to affective valences^42^. PD patients experience negative feelings such as fluctuations in personal competence, low self-confidence and a sense of shame due to their unwilling psychical symptoms, which are further connected to social withdrawals^34^. Actions made by the limb with stronger motor impairment may therefore lead to negative valences such as less confidence, resulting in weakened action-effect association. Priors and beliefs of action are other factors to influence personal authorship^35, 36^. A prior knowledge about the side of stronger motor impairment may lead to change in awareness of action. Because there was no significant difference in AB between the more and the less affected side of the hand in PD, central sensorimotor processing could have mattered more than these psychological effects following motor impairment. The results of the implicit task indicate that PD patients experience a change of action awareness in the non-conceptual level, with strong involvement of the sensorimotor domain.

In the explicit task, PD patients showed less attribution of the given feedback to themselves compared to the control group in the non-biased and angular biased conditions. Here, attribution of movements in the more and the less affected hand also did not differ. PD patients largely depend on external cues for compensation. Providing external visual cues will improve motor impairments such as bradykinesia^43^. It has also been reported that, when patients cannot see their movement hand, they undershoot the movement targets^44^. Although visual and kinesthetic inputs play roles in sensory feedback, patients largely rely on visual cues. In our experiment, participants’ actual movements of their own hands were concealed, making it difficult for the patients to predict spatial trajectory of movements in angular biased condition. In contrast, kinesthetic inputs should be sufficient to predict the consequence of movement initiation in temporal biased condition. This may have led to different results in angular and temporal conditions. Another explanation for the dissociation between angular and temporal conditions might also be possible. Because PD patients usually experience a time lag of their own movement as part of bradykinesia, and the lag is in a single direction, namely, a delay, compensation could have occurred, or it may have been easier for patients to predict action consequence in temporal condition than in angular condition. These results of the explicit task indicate that PD patients also experience a change in the conceptual judgement level for attribution of action.

It has been suggested through neurophysiological observations that changes of sensorimotor integration in PD do not take place at the peripheral level but depend on central processing of sensory input^5^. Studies show that non-motor symptoms in PD originate from bilateral pathological changes of widespread regions of the brain, starting from an ascending involvement of the lower brain stem nuclei, as well as the cerebral cortex^45^. Non-motor symptoms in PD can precede the occurrence of motor features, both clinically and pathologically^46, 47^. It was shown that abnormal motor- and cognition-related brain networks activity was evident bilaterally in hemiparkinsonian patients before clinical onset on the opposite body side^39^. Diverse brain regions play roles to form the awareness of action^25^. The observed changes might be related to the widespread pathological progression in PD, although further investigation would be needed.

A limitation of this study is that patients participated in the experiment while under regular dopaminergic medication. This could be a confounding factor for the results. However, total daily LED and the performances of the two experiments (bindings in Experiment 1 and total number of “yes” responses in Experiment 2) did not show significant correlation. In addition, it has been shown that intakes of dopamine strengthen the causal association of action and its effects^27^. Regarding these points, our results can be interpreted such that the patients showed less causal action-effect association even though medication worked in the opposite direction. Succeeding studies excluding the effects of medication would be needed for further interpretations. The small sample size of both PD patients and healthy controls is another limitation of this study. Employing only two of the representative measures among various means of assessing the awareness of action can be another limitation. Examinations by various means reflecting the volitional control of action are needed for further discussion.

In summary, we investigated the awareness of voluntary action in PD through implicit and explicit tasks. In both tasks, PD patients showed different patterns of reaction from controls, suggesting change in the subjective awareness of action in PD. These tendencies were consistent regardless of the side of asymmetrical motor symptoms, supporting a linkage to primary deficits in central sensorimotor processing rather than to secondary effects from motor impairment. Further investigations will be required to examine the underlying neurobiological basis.

## Methods

### Participants

Nineteen patients with PD (eight females) and twenty-five normal control subjects (thirteen females) participated in the study. Patients were recruited from Kyoto University Hospital and met the UK Parkinson’s Disease Society Brain Bank Clinical Diagnostic Criteria. Patients were excluded if they had a cognitive decline that prevented them from providing informed consent. The Hoehn and Yahr stages of the patients were 1–3. Eighteen patients were being treated with daily dopaminergic medications. Patients were on their daily medication at the time of the experiment. The total daily levodopa equivalent dose (LED) was calculated^48^. Sixteen patients had left dominant onset of the disease, while three patients had right dominant onset. Motor symptoms were assessed with the UPDRS Part 3 motor subscale. The UPDRS right and left subscales were calculated by combining the scores of the UPDRS part 3 items 20–26 in each of the right and left limbs. The more-affected and the less-affected sides of motor symptoms were decided by comparing these scores, as right symptom dominant (right > left) and left symptom dominant (left > right). None of the controls had a known neurological or psychiatric history. All participants in both groups were right-handed according to the Edinburg Inventory^49^. BDI^50^, Apathy Scale^51^, General Self Efficacy Scale^52, 53^, Cognistat^54^, and Frontal Assessment Battery (FAB)^55^ were assessed in both groups. Written informed consent was obtained from each participant. This study was approved by the Kyoto University Graduate School and Faculty of Medicine Ethics Committee and was carried out in accordance with The Code of Ethics of the World Medical Association.

### Experiment 1- implicit task

#### Procedures

The sequence of events from a previous study^22^, known as intentional binding task, was employed. The task consisted of four categories of conditions: 1) agency action, 2) agency tone, 3) baseline action, and 4) baseline tone. In the agency action and agency tone conditions, participants performed a voluntary action. In each condition, a blank screen was first presented, followed by a picture of a clock-face and clock-hand. The clock-hand was 12 mm long, and it rotated clockwise for a full rotation in 2,560 ms. The clock-face was marked with 12 conventional interval positions (5, 10, 15, etc.). Initial positions of the clock-hand were chosen randomly from the 12 positions. The clock-hand remained stationary at the initial position for 500 ms, and then began to rotate. Participants performed a key press at a time of their own choosing during clock-hand rotation. They were instructed to avoid responding at a pre-decided clock position, or during the first half-rotation of the clock-hand. Each key press triggered a tone after a fixed period of 250 ms. The tone duration was 100 ms, and the frequency was 1000 Hz. In the agency action condition, participants were asked to report the perceived onset time of their voluntary key press as judged by the perceived position of the clock-hand. Similarly, in the agency tone condition, participants were asked to report the perceived onset time of the triggered tone. In the baseline action condition, participants performed a voluntary key press at the time of their own choosing, but this did not yield a tone. Participants reported the perceived onset time of their voluntary key press. In the baseline tone condition, participants did not press a key but instead waited for a tone to be delivered, judging the onset time at which they heard the tone. Because the performance of patients could be disturbed by motor symptoms, trials that were missed or not conducted successfully were repeated. The first 24 trials conducted successfully with the participants’ intended button press were brought to analysis. After completing the task with one hand, participants conducted the task with the other hand. The order of right and left hand was counterbalanced across the participants. In order to keep participants naïve to the purpose of the experiment, the implicit task was conducted first, followed by the explicit task. Details of the task procedure are described elsewhere^32^.

#### Data analysis

The perceived time of action or tone in each trial was compared with the actual onset time. The mean estimation for actions and tones in the baseline condition was subtracted from that in the agency condition. These shifts served as measures of “action binding (AB)” and “tone binding (TB)”, respectively.

AB and TB, respectively, were examined by mixed-effect ANOVA, with group (patients versus controls) as between-subject factor, and hand (more-affected versus less-affected hand in patients, left versus right hand in controls) as within-subject factor. The more-affected hand in patients was matched to the left hand in controls, and the less-affected hand in patients was matched to the right hand in controls for the ANOVA analysis, as there were more patients with left dominant onset than with right dominant onset and prognosis of the disease.

### Experiment 2- explicit task

#### Procedures

A simplified task from a previous study^21^ was employed. Participants were asked to hold a joystick that was connected to a computer. A black cover was placed over the joystick so that the participants could not see their actual movement. Instead, an image of an electronically constructed virtual hand was presented to the participants on a computer screen. Participants were instructed that “their hand” would appear on the computer screen. Participants made voluntary movements of the joystick, while a program synthesized images of a virtual hand holding a joystick and the virtual hand moved according to the position that was actually held by the participants. The movement of the joystick was presented on the screen with an intrinsic delay of 16 ms. In each trial, after a blank screen, an image of a virtual hand was presented for 10 sec, during which participants were asked to move the joystick according to their own choosing. Movement could be executed in four directions (right, left, back, and forth). A yes-or-no question of correspondence was made after participants stopped their movement. Participants were asked a yes-or-no question as follows: “Did the movement you saw on the screen correspond to the movement you made with your hand?” Participant’s hand was back in the home position when answering the question.

The task consisted of three categories of conditions: 1) neutral, 2) with angular biases, and 3) with temporal biases. In the neutral condition, the virtual hand moved exactly according to the movements the participants made with the joystick. In the angular biases condition, a given angular value (5°, 10°, 15°, or 20°) was introduced, and in the temporal biases condition, a given time delay (50, 100, 150, 200, 300, 400, or 500 ms) was introduced as a gap between the movements of the virtual hand and the joystick. Trials with angular biases and trials with temporal biases were run four times for each type of gap. Neutral trials were run twelve times. The order of presentation of all trials was randomized for each subject. Before running the experiment, participants performed a practice session. Missed trials were repeated. After completing the task with one hand, participants conducted the task with the other hand. The order of right and left hand was counterbalanced across participants.

**Figure 3:**
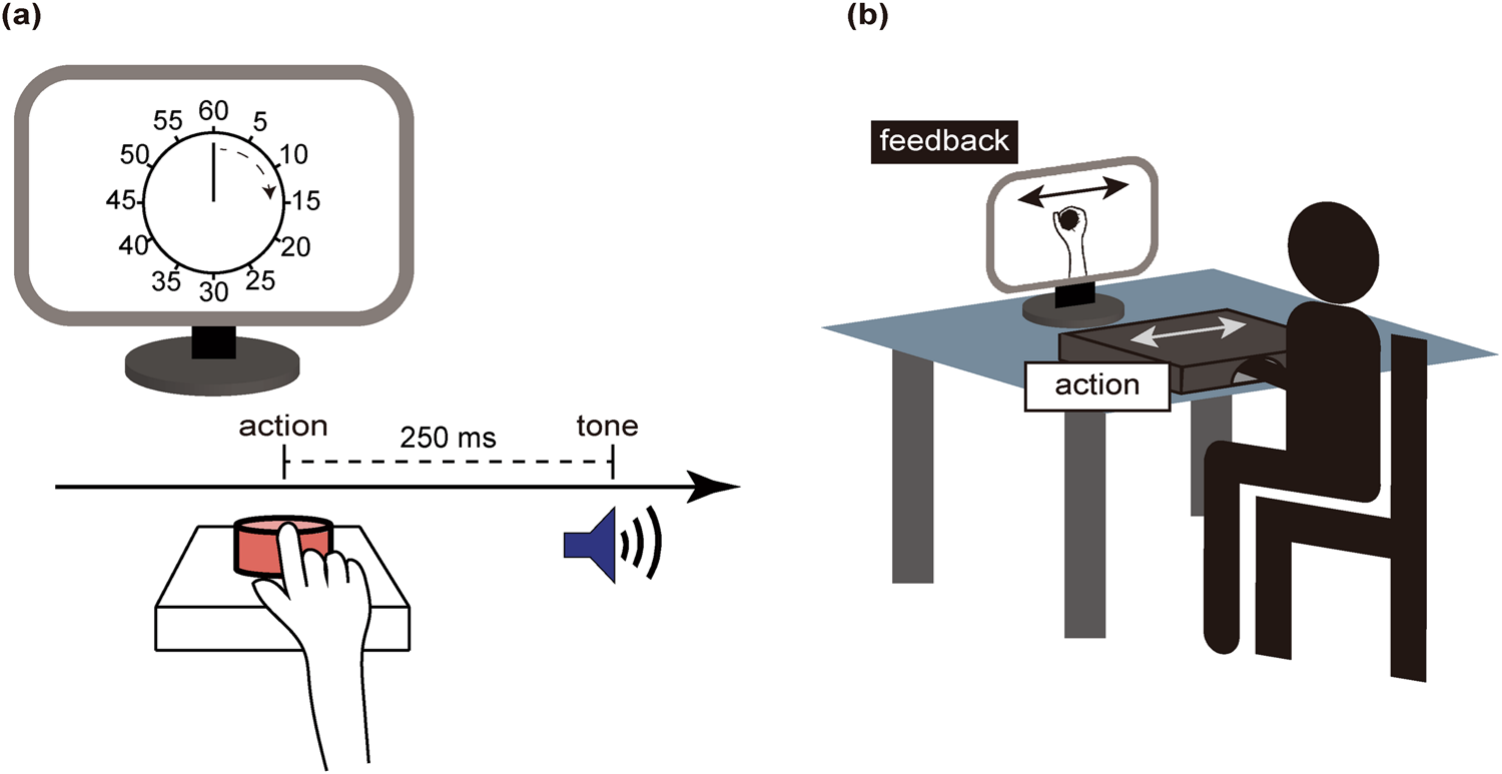
Illustration for experimental setups and protocols for (**a**) Experiment 1 and (**b**) Experiment 2.

There could potentially be two types of errors in the reaction: “yes” responses for trials with biases, and “no” responses for neutral trials. For data analysis, “yes” responses were focused upon, reflecting the participants’ ability to recognize the movement as their own. The experimental setups are illustrated in Fig. 3.

#### Data analysis

One patient was excluded from the analysis due to a technical failure in presenting the task. The percentage of ‘yes’ responses in the angular biases condition was analyzed by mixed-effect ANOVA, with group (patients versus controls) as between-subject factor, and event (gap of 0°, 5°, 10°, 15°, or 20°) and hand (more-affected versus less-affected in patients, left versus right in controls) as within-subject factor. Next, the percentage of ‘yes’ responses in the temporal biases condition was analyzed by mixed-effect ANOVA, with group (patients versus controls) as between-subject factor, and event (delay of 0, 50, 100, 150, 200, 300, 400, or 500 ms) and hand (more-affected versus less-affected in patients, left versus right in controls) as within-subject factor. Degrees of freedom were adjusted by Greenhouse-Geisser correction. The more-affected hand in patients was matched to the left hand in controls, and the less-affected hand in patients was matched to the right hand in controls in the following analysis, for the same reason as in implicit task.

## Electronic supplementary material

Supplementary Table S1
