# Supplementary material for: Altered awareness of action in Parkinson’s disease: evaluations by explicit and implicit measures
Source: Sci Rep. 2017 Aug 14;7:8019. doi: 10.1038/s41598-017-08482-0 (PMC5556120; doi:10.1038/s41598-017-08482-0)
Supplement: Supplementary file 1 — Supplementary Table S1 [file 41598_2017_8482_MOESM1_ESM.pdf]

Altered awareness of action in Parkinson's disease:
evaluations by explicit and implicit measures

Naho Saito, Keisuke Takahata, Hodaka Yamakado, Nobukatsu Sawamoto,
Satoshi Saito, Ryosuke Takahashi, Toshiya Murai, Hidehiko Takahashi*

* Correspondence should be addressed to Hidehiko Takahashi,
Department of Psychiatry, Kyoto University Graduate School of Medicine,
54 Shogoin-Kawahara-cho, Sakyo-ku, Kyoto 606-8507, Japan
Tel: +81-75-751-3386, Fax: +81-75-751-3246
E-mail: hidehiko@kuhp.kyoto-u.ac.jp

Supplementary Table S1

	Experiment 1		Experiment 2	
	Action Binding	Tone Binding	Angular condition	Temporal condition
**Parkinson's Disease patients**				
Age	0.39 (0.09)	-0.39 (0.10)	-0.04 (0.87)	0.20 (0.43)
UPDRS motor subscale	0.04 (0.88)	-0.12 (0.63)	0.20 (0.43)	0.18 (0.49)
Total daily LED	0.20 (0.41)	0.01 (0.97)	0.04 (0.87)	0.00 (1.00)
BDI	-0.14 (0.58)	-0.22 (0.36)	0.33 (0.19)	0.43 (0.07)
Apathy Scale	0.04 (0.88)	0.13 (0.60)	-0.14 (0.58)	-0.15 (0.56)
General Self Efficacy Scale	0.07 (0.77)	-0.37 (0.12)	0.11 (0.66)	0.16 (0.54)
Cognistat	-0.23 (0.34)	-0.05 (0.84)	-0.18 (0.48)	-0.16 (0.53)
**Controls**				
Age	-0.28 (0.17)	0.07 (0.75)	-0.07 (0.75)	0.22 (0.29)
BDI	0.21 (0.31)	-0.33 (0.11)	0.11 (0.60)	-0.09 (0.68)
Apathy Scale	-0.14 (0.51)	-0.17 (0.41)	0.19 (0.36)	0.12 (0.56)
General Self Efficacy Scale	0.21 (0.31)	0.20 (0.33)	-0.01 (0.95)	-0.28 (0.18)
Cognistat	0.03 (0.90)	0.02 (0.91)	-0.12 (0.57)	-0.38 (0.06)

Correlations between performances of the experiments (Action/Tone Binding in Experiment 1, total number of "yes" responses in the two conditions in Experiment 2) and covariates by Spearman rank correlations. Correlation coefficients (ρ) are shown in the table. *P* values are shown in parentheses.
